# Schrödinger's tree—On syntax and neural language models

**DOI:** 10.3389/frai.2022.796788

**Published:** 2022-10-17

**Authors:** Artur Kulmizev, Joakim Nivre

**Affiliations:** ^1^Computational Linguistics Group, Department of Linguistics and Philology, Uppsala University, Uppsala, Sweden; ^2^RISE Research Institutes of Sweden, Kista, Sweden

**Keywords:** neural networks, language models, syntax, coding properties, representations, natural language understanding

## Abstract

In the last half-decade, the field of natural language processing (NLP) has undergone two major transitions: the switch to neural networks as the primary modeling paradigm and the homogenization of the training regime (pre-train, then fine-tune). Amidst this process, language models have emerged as NLP's workhorse, displaying increasingly fluent generation capabilities and proving to be an indispensable means of knowledge transfer downstream. Due to the otherwise opaque, black-box nature of such models, researchers have employed aspects of linguistic theory in order to characterize their behavior. Questions central to syntax—the study of the hierarchical structure of language—have factored heavily into such work, shedding invaluable insights about models' inherent biases and their ability to make human-like generalizations. In this paper, we attempt to take stock of this growing body of literature. In doing so, we observe a lack of clarity across numerous dimensions, which influences the hypotheses that researchers form, as well as the conclusions they draw from their findings. To remedy this, we urge researchers to make careful considerations when investigating coding properties, selecting representations, and evaluating *via* downstream tasks. Furthermore, we outline the implications of the different types of research questions exhibited in studies on syntax, as well as the inherent pitfalls of aggregate metrics. Ultimately, we hope that our discussion adds nuance to the prospect of studying language models and paves the way for a less monolithic perspective on syntax in this context.

## 1. Introduction

Syntax—how words are combined to form sentences in natural language—has perhaps never garnered as much attention from NLP researchers as it does in the present day. Naturally, its recent relevance at conferences is owed to the deep learning paradigm, which the NLP community has embraced with open arms since the midpoint of the last decade. Prior to this paradigm shift, questions central to syntax were often restricted to the parsing domain. There, researchers were largely interested in developing supervised algorithms for processing structured input—usually in the form of annotated constituency or dependency treebanks. Beyond parsing, syntax also often factored into researchers' hypotheses about what information models may need to succeed in a given task. Feature engineering was a pivotal component of pre-neural NLP, where text was filtered through hand-crafted feature templates that emphasized parts of speech, morphology, and tree structure, so as to inform simple, often linear models about the underlying syntax of sentences.

The deep learning revolution of the mid 2010s quickly obviated the need for feature engineering, which was widely considered a time-consuming and painstaking process. Embeddings—dense vectors representing the distributional properties of words—quickly replaced the sparse, hand-crafted vectors of yore and boosted performance dramatically (Mikolov et al., 2013; Pennington et al., 2014). Such progress presented a trade-off, however: accuracy at the expense of interpretability. Indeed, without the guiding hand of the feature engineer, it became difficult to ascertain what properties of natural language the new neural models—highly complex and non-linear—had come to rely on.

It was this uncertainty that inspired a new line of inquiry within NLP, concerning what exactly models know and how they come to learn it. Early insights from this domain intimated that neural networks could capture facets of the hierarchical structure of language, beyond the linear order of words in a sentence. The Long Short Term Memory network (LSTM) (Hochreiter and Schmidhuber, 1997) featured prominently in such studies, where researchers employed linguistic minimal pairs (mostly based on agreement phenomena) in order to demonstrate the model's sensitivity to syntactic hierarchy (Linzen et al., 2016; Gulordava et al., 2018). Such findings were deemed exciting mainly due the LSTM's design as a sequence processor, which lacked the sort of structural supervision or inductive bias that one might encounter in the parsing literature.

Amidst skyrocketing research budgets and the continued advancement of processing hardware, NLP faced another paradigm shift in 2018–2019. Researchers began realizing that representations for input words need not be fixed to a single static vector per type (as with word embeddings), but can instead be computed dynamically, with each word contextualized with respect to the rest of the sentence (Peters et al., 2018). Per this logic, it also became apparent that models capable of generating such representations could be fine-tuned with respect to downstream tasks, with impressive gains in performance thereafter (Howard and Ruder, 2018). Language models—the basis of classic word embedding algorithms—were a natural fit for this paradigm and became NLP's backbone going forward.

In the modern day, models like BERT (Devlin et al., 2019), GPT (Radford et al., 2019), and their successors feature prominently in NLP research, showcasing the efficacy of the pretrain-and-finetune paradigm. Naturally, the human-like generation capability of such models, as well as their success on natural language understanding (NLU) benchmarks (Wang et al., 2018, 2019), makes the question of what the models know about language and how they acquire such knowledge and ever-pressing one. Increasingly, we find, NLP researchers turn to the field of syntax—with its decades of research, theory, and debate—in order to answer such questions. In this paper, we attempt to take stock of the ever-growing literature on the syntactic capabilities of neural language models. In doing so, we observe a lack of clarity across numerous dimensions, which influences the hypotheses that researchers form, as well as the conclusions they draw from their findings. We argue that this failure of articulation results in a body of work whose hypotheses, methodologies, and conclusions comprise many conflicting insights, giving rise to a paradoxical picture reminiscent of Schrödinger's cat—where syntax appears to be simultaneously dead and alive inside the black box models. In particular, by framing studies around aggregate metrics and benchmarks, syntax is often reduced to a monolithic phenomenon, which fails to do justice both to the complex interplay between different manifestations of hierarchical structure in natural language and to the substantial variation that exists across typologically different languages.

Our goal in this article is not to criticize earlier studies, which all provide valuable pieces of evidence for understanding the role of syntax in contemporary NLP, particularly language models. Instead, we propose a number of conceptual distinctions, the consideration and articulation of which, we argue, can help us better understand the seemingly conflicting results, resolve some of the apparent contradictions, and pave the way for a more nuanced and articulated research agenda. To provide the necessary background for this analysis, we begin by introducing the concept of syntax from a bird's eye perspective. We then review a representative sample of investigations into the syntactic capabilities of neural language models, which we categorize as belonging to three different paradigms. We supplement this review by discussing what we perceive to be important distinctions about syntax left implicit in this body of work. This leads to a discussion of different classes of research questions underlying the surveyed literature, and the role of aggregate metrics in addressing these research questions. We conclude with some thoughts on how our analysis can inform our research methodology for the future.

## 2. Background: Aspects of syntax

Syntax is usually described as the way that words are combined into larger expressions like phrases and sentences. On one hand, syntax can then be contrasted with morphology, which is concerned with the internal structure of words. On the other hand, it can be contrasted with semantics, which deals with the *meaning* of words, phrases and sentences—as opposed to their *form*. In reality, however, syntax is concerned with the complex mapping between *form* and *meaning* at the phrase and sentence level. It is therefore important to make a distinction between *syntactic structure*—an abstract hierarchical structure that determines or constrains semantic composition—and *coding properties*—expressive devices such as word order, function words and morphological inflection that are used to partially encode the syntactic structure. To illustrate this point, let us consider two equivalent sentences in Finnish and English:


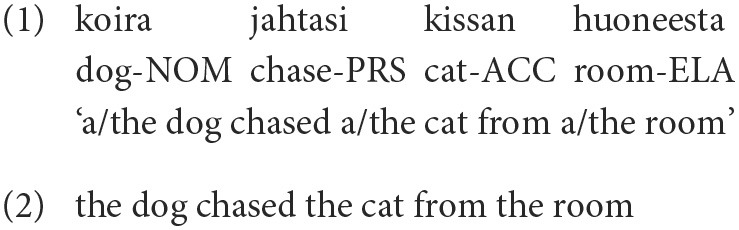


Most linguists would agree that (1) and (2) not only mean (roughly) the same thing but also have a similar syntactic structure, where the main verb (*jahtasi, chased*) takes a subject (*koira, the dog*), a direct object (*kissan, the cat*) and a locative modifier (*huoneesta, from the room*). However, the encoding of this syntactic structure is quite different in the two languages. In English, the subject and object are primarily identified through their position relative to the verb, while the locative modifier is introduced by a preposition (*from*). In Finnish, the role of all three dependents of the verb is indicated by morphological case inflection, and constituent order is not significant^1^. Note also that the overt coding properties (word order, function words, morphological inflection) do not (always) uniquely determine the syntactic structure. For example, in the English example, the phrase *from the room* could also function as a modifier of the noun phrase *the cat*, although this is a less likely interpretation in most contexts.

While coding properties are concrete aspects of the sentence, the syntactic structure is essentially an abstract concept that is not directly observable. Nevertheless, linguists have over the years accumulated compelling evidence for the existence of a hierarchical structure over and above the sequential order of words. The most obvious type of evidence is perhaps the occurrence of structural ambiguity, where a single sequence of words can be assigned multiple interpretations, exemplified in the following classic examples:

(3) she saw the man with the telescope
(4) old men and women
(5) flying planes can be dangerous

The principle of compositionality states that the meaning of a complex expression is determined by the meanings of its constituent expressions and the rules used to combine them. Since the different interpretations in the examples above are not due to lexical ambiguity, they must be due to the rules used to combine the constituent expressions. Hence, they show that different syntactic structures can be realized by the same sequence of words. According to this view, the abstract syntactic structure is closely connected to semantic composition and the syntax-semantics interface. Other types of evidence for a hierarchical syntactic structure come from substitution and permutation tests (see, e.g., Matthews, 1981).

While the existence of a hierarchical structure is hardly contested today, the linguistic theories developed to account for this structure vary in their theoretical assumptions as well as in their mathematical representations of syntactic structure. The generative grammar tradition has been dominated by theories based on phrase structure (constituency) (Bloomfield, 1933; Chomsky, 1957), with successively more abstract representations. An alternative conception of syntax is found in theories based on dependency structure (Tesnière, 1959; Mel'čuk, 1988), which emphasize the functional role of linguistic expressions over their constituent structure. A third theoretical tradition is that of categorial grammar (Ajdukiewicz, 1935; Steedman, 2000), which is based on combinatory logic and assumes a close connection between syntax and semantic composition. To some degree, it is possible to convert syntactic representations from one theoretical framework to another, but the conversion is usually heuristic and lossy and, therefore, the different representations are not commensurable, strictly speaking.

The existence of a wide range of syntactic theories arises from contested views on how a diverse range communicative principles, including the use of different coding properties, can come to exist across languages. For example, the Chomskyan tradition posits that an innate human grammar—a set of rules and processes that govern human cognition—is privy to a series of language-specific transformations that result in such idiosyncrasies (Chomsky, 1965, 1981, 1995). Other accounts argue that syntax itself is shaped by functional or cognitive constraints (Zipf, 1949; Givón, 1995; Hawkins, 2004; Jaeger and Tily, 2011; Gibson et al., 2019), such as managing memory load by preferring dependencies of shorter length (Gibson, 1998; Gibson et al., 2000)—a process which can also influence coding properties like word order (Futrell et al., 2020; Hahn et al., 2020). Cultural differences across languages are likewise theorized to play a large role (Evans and Levinson, 2009;), with complex morphosyntactic processes like polysynthesis being largely observable in small, non-industrial communities with dense social-network structures (Trudgill, 2017). Directly or not, such debates revolve around the controversial *poverty of the stimulus* argument (Lasnik and Lidz, 2017)—linguistics' own spin on psychology's nature vs. nurture debate—where the human capacity to acquire and generalize across structures is perceived as either predominantly learned or predominantly innate.

Neural networks—especially large scale language models—have recently assumed an interesting place in this discussion. Primarily, syntactic theory has offered a useful toolkit for more fine-grained evaluation of language models, which have shown an ability to generate coherent, grammatical output, resembling that of humans. To this end, researchers have employed well-studied coding properties like subject-verb agreement (Linzen et al., 2016) or phenomena like filler-gap dependencies (Wilcox et al., 2018) to articulate exactly on which grounds a models' output might be judged as grammatical or not. Such studies have served as a welcome complement to the ubiqutious, yet opaque perplexity metric—a measure of how predictable sentences or documents are, given a model's parameterization. In a sense, however, they can likewise be perceived as a means of *sanity-checking* models' behavior (Baroni, 2021), with paper titles often framed interrogatively: *Do neural language models learn* _____? Nonetheless, answering such questions is useful, and a concrete understanding of the ability of neural networks to generalize with respect to natural language—as well as the algorithmic processes that underlie this capacity—could, in the least, provide interesting perspectives on the age-old debates mentioned above (Linzen and Baroni, 2021).

## 3. Review: The quest for syntax

In this section, we review work belonging to what we perceive as the three dominant paradigms for attesting language models' knowledge of syntax—targeted syntactic evaluation, probing, and (downstream) NLU evaluation. Though comprehensive surveys of such studies can be found, for example, in Linzen and Baroni (2021) or Manning et al. (2020), our aim here is to relate them to the concepts and distinctions discussed in the previous section. Readers interested in a more detailed description and analysis are referred to the aforementioned work.

### 3.1. Targeted syntactic evaluation

Targeted syntactic evaluation^2^ (TSE) is arguably the most popular framework for assessing neural networks' ability to make syntactic—therefore hierarchical—generalizations. At its core, TSE is a black-box testing approach concerned with measuring model output (typically probabilities) with respect to a curated set of stimuli. Such stimuli are typically based on minimal pairs motivated by phenomena in the syntax literature. For example, consider the (by now classic) example in sentences 6a and 6b.

(6) a. The keys to the cabinet are on the table.
b. *The keys to the cabinet is on the table.
c. *The key to the cabinets are on the table.
d. The key to the cabinets is on the table.

The literature dictates that a competent English speaker would rely on a structural analysis of *the keys to the cabinet* to infer number agreement between the plural subject (*keys*) and the copula verb (*are*). On the other hand, a purely sequential processing of the sentence would arrive at the opposite conclusion in 6b: *is* agrees with the adjacent singular noun (*cabinet*). To ascertain whether or not a language model *M* follows the former logic, one could, for example, compare the probabilities assigned to the target verb *be* in 6a–6b, given the context *C*= *the keys to the cabinet*, and examine whether *P*_*M*_(are|*C*)>*P*_*M*_(is|*C*). This can also be extended to full paradigms, where, in the case of 6, *M* has to assign higher probabilities to both (6a) and (6d) with respect to (6b) and (6c). TSE (per this formulation) can thus be seen as based on an accuracy metric, which, if returning a high value over *n* stimuli, implies that *M* is able to generalize with respect to the relevant syntactic phenomenon. It should be noted that probability assigned to the word form *x*, per various theoretical justifications, is sometimes replaced with surprisal, e.g., *S* = −log_2_*P*_*M*_(*x*|*C*), as in Wilcox et al. (2018) and Futrell et al. (2019). Furthermore, in situations where the locus of ungrammaticality does not lie on a single word (as in English subject-verb agreement), but is dependent on the interaction of several words (e.g., as in negative polarity items), it is common to compare the probabilities or perplexities of entire sentences (Jumelet and Hupkes, 2018; Marvin and Linzen, 2018).

The TSE framework also allows for flexibility in integrating more complex sets of stimuli, as in the study on syntactic state by Futrell et al. (2019):

(7) a. As the doctor studied the textbook, the nurse walked into the office.
b. *As the doctor studied the textbook.
c. ?The doctor studied the textbook, the nurse walked into the office.
d. The doctor studied the textbook.

With respect to (7), Futrell et al. (2019) formulate a set of hypotheses, whereby they posit (1) that the surprisal at the matrix clause after the comma (... *the nurse walked into the office*.) should be lower for (7a) than for (7c) (the network knows it is in a subordinate clause per the subordinator *as*), and (2) that the surprisal at the matrix clause should be higher for 7b than 7d (the network expects a matrix clause per the subordinator). Though the aforementioned accuracy approach could likewise be appropriated here as a summary statistic, researchers also often employ significance testing in order to accept or reject their hypotheses. For example, Futrell et al. (2019) apply a linear mixed-effect model on their models' stimulus-level predictions in order to accept hypothesis (1) on behalf of all models, but reject hypothesis (2) for all but two. This formulation—in line with common paradigms in psycholinguistics—leads them to conclude that, while all models are partially capable of tracking syntactic state across subordinate and main clauses, certain training conditions are required (large data or explicit structural objectives) in order to fully capture the structural expectations induced by subordinators. A similar methodology is employed in Wilcox et al. (2018) for investigating filler-gap dependencies.

The popularity of the TSE framework has precipitated the creation of challenge suites, which offer holistic measures of models' performance across a variety of linguistic phenomena. Marvin and Linzen (2018) were among the first to introduce such datasets, employing a context-free grammar to procedurally generate minimal pair sentences—such as 6a and 6b—for a variety of phenomena: agreement (of various kinds), reflexive anaphora, and negative polarity items. Warstadt et al. (2020) later presented a similar, automatically generated dataset of minimal pairs (BLiMP), albeit with wider coverage: 1,000 sentences per 67 paradigms belonging to 12 different phenomena. The authors used BLiMP to study various popular language model architectures (LSTM, Transformer), whereby they associated average accuracy across phenomena with models' linguistic knowledge. A similar suite was contemporaneously introduced by Hu et al. (2020), albeit in employ of 2 × 2 templates like 6 for hand-curated stimuli culled from syntax textbooks. Like Warstadt et al. (2020), Hu et al. (2020) used their suite^3^ to study language model architectures, most notably relating language models' syntactic generalization (SG) score—measured in aggregate across phenomena—to their test set perplexity.

It is important to note that the aforementioned datasets and challenge suites are primarily designed to evaluate the syntactic knowledge of *pre-trained* models. Indeed, there exists a parallel line of work that aims at clarifying the generalization capacity of popular architectures (such as LSTMs or Transformers) when trained *from scratch* on curated—often grammar-generated—data. One such dataset is COGS (Kim and Linzen, 2020)—a semantic parsing dataset constructed in such a way that the evaluation (or generalization) set contains combinations of lexical items and syntactic structures that do not occur in the training set. In COGS, sequence-to-sequence models trained on sentences where certain lexical items occur, for example, only in subject position (*a hedgehog ate the cake*) must generalize over structural word order patterns when the same lexical items appear in the object slot (*the baby liked the hedgehog*). Another such dataset is CFQ (Keysers et al., 2019), which tests models' ability to parse natural language into SPARQL when the distribution of compositional rules across train and test are purposefully divergent. In both cases, as well as many others (see Baroni, 2020 for an overview), it has been shown that out-of-the-box models like LSTMs and Transformers dramatically fail to generalize to samples outside of their training distributions (though specialized architectures can do so trivially). For example, Kim and Linzen (2020) report that Transformers and Bi-LSTMs yield meager average accuracies of 0.31 and 0.05, respectively, on the Subject → Object rule described above. Though it must be acknowledged that such setups differ from TSE in targeting cold-started seq2seq models rather than pre-trained language models, and employing synthetic rather than naturalistic data, they are similar in that they study model responses to controlled stimuli. Moreover, their focus on the compositional aspects of syntax makes them an interesting alternative approach that may shed light on some of the potential confounds potentially associated with TSE.

### 3.2. Probing

Probing^4^ is another popular paradigm for attesting NLP models' acquisition of syntax. The key distinction between TSE and probing is that, while the former is concerned with model behavior, the latter focuses explicitly on model *representation*. In this context, behavior is likened to the probabilities assigned to certain outputs (extracted, typically, from the output layer of a language model), while representation refers to the intermediate hidden state vectors computed by the model. Mainly, probing is motivated as being necessary due to deep learning's end-to-end nature: features are learned with respect to a given task, not engineered like in traditional systems. Due to this fact, neural models' representations are wholly uninterpretable to the human interlocutor and thus require intervention in order to understand what they portray.

Formally speaking, probing is concerned with representations **h** extracted from a model *M* for a given input *x*: **h** = *M*(*x*). A representation **h**∈ℝ^1 × *d*^ is typically a fixed-length dense vector corresponding to input word *x* (e.g., *keys* in 6a), where *d* is the hidden-state dimensionality of *M*. A probe *f* for a given linguistic property *A* is a classifier fit on **h** to produce output *y*∈*Y*, where *Y* is a finite label set: *y* = *f*_*A*_(**h**). For properties that can be decoded from single words, such as part-of-speech (POS) tags, a trained probe *f*_POS_ must be able to assign the correct label to **h** with respect to the ground truth, e.g., ŷ = NOUN for *M*(keys). For properties concerning two or more words, such as dependencies or phrases, a concatenation of hidden states corresponding to (possibly) discontiguous tokens *x*_*i*_, *x*_*j*_ or a contiguous span of tokens *x*_*i*_, ..., *x*_*j*_ is applied. In this latter formulation, deemed edge-probing by Tenney et al. (2019b), one might expect a probe *f*_DEP_ to decode ŷ = NSUBJ for *M*(keys, are) and *f*_CON_ to decode ŷ = PP for *M*(on, the, table). Though probing models vary widely in terms of architecture, parameters, optimization, etc., the vast majority of them assume a training set *D*_*A*_ representative of property *A* on which *f*'s parameters Θ can be fit, like a treebank. Such probes are then evaluated in standard supervised learning fashion *via* accuracy on a held out test set. If such accuracy is high, it can then be said that *A* is decodable from **h**, i.e., that *M* learns it. This framework was notably employed by Liu N. F. et al. (2019) and Tenney et al. (2019b), who concurrently demonstrated that representations extracted from popular contextual embedding models (ELMo, BERT, GPT) yielded exceedingly good performance on suites of linguistic tasks. Also noteworthy is Tenney et al. (2019a)'s study, which showed that BERT's representations appear to evolve in capturing properties with increasing levels of complexity, from lexical features to syntax and semantics.

While the aforementioned word-level approach is by far the most popular probing setup, other methods for decoding the syntactic structure of entire sentences have been proposed. One model that is of particular interest is that of Hewitt and Manning (2019), who attempt to decode dependency structure from models' vector spaces. To this end, they propose to learn transformations over model representations, such that (1) the squared *l*_2_ distance between any vectors **h**_*i*_, **h**_*j*_ reflects the distance between their corresponding words *x*_*i*_, *x*_*j*_ in a parse tree, and (2) that the *l*_2_ norm of any vector **h**_*i*_ reflects the depth of its corresponding word *x*_*i*_ in a parse tree. They find that this method is particularly effective for decoding Stanford Dependencies trees (de Marneffe et al., 2006) from ELMo and BERT representations, with respect to several lexical-only baselines. Beyond Hewitt and Manning (2019)'s method, which can be imagined as doing parsing by proxy, other work has directly employed (underparameterized) dependency parsers as probes. For example, Hewitt and Liang (2019) employ a graph-based bilinear probe; Maudslay et al. (2020) investigate the relation between probing and parsing; and Pimentel et al. (2020a) advocate for adding full dependency parsing to the probing task suite. A potential advantage of probes that attempt to decode the syntactic structure of a complete sentence is that they may shed light on the compositional aspects of syntax—as well as a model's encoding thereof—in a way that is complementary to the studies based on synthetic data discussed in Section 3.1.

At this stage, probing can be considered a field of inquiry in its own right, with researchers presenting new models, metrics, and criticisms for every conference cycle. Naturally, the use of intermediary models trained on top of extracted representations warrants caution from the interlocutor. Concerns expressed in the literature include but are not limited to the following: the use of smaller, linear models vs. larger, nonlinear ones; appropriate baselines and evaluation metrics; properties being learned by the probe vs. occurring in representations; properties being employed by the model in the original task vs. simply being decodable, etc. Though a full consideration of these methodological concerns is outside the scope of this article, we refer the interested reader to Belinkov (2022)—a comprehensive review of the paradigm, open issues, and alternative approaches like attention analysis.

### 3.3. NLU evaluation

Outside of TSE and probing, another technique that has recently attracted much attention is the evaluation of models (imbued with or deprived of syntactic knowledge) on downstream tasks. The logic inherent to this line of inquiry is as follows: if a model has come to rely on human-like knowledge of language (or some semblance thereof) to solve complex NLP tasks, then it should (1) perform *poorly* on such tasks when the surface form of an utterance has been corrupted beyond (human) comprehension, and (2) perform *better* when imbued with the exact abstract structure theorized by linguists as governing the surface form. Such tasks are typically taken from the GLUE benchmark—a suite of natural language understanding (NLU) datasets “designed to favor and encourage models that share general linguistic knowledge across tasks” (Wang et al., 2018). GLUE has served as the principal point of comparison for pretraining architectures, where, as of writing, 15 models have surpassed the published human performance on the same tasks.

In terms of input corruption, many studies have investigated the effect of word order on NLU task performance. Indeed, word order is the primary means of encoding syntactic argument structure in English, and such work often hypothesizes that sensitivity to this particular property should result in lower NLU scores. Gupta et al. (2021) demonstrate that this is not the case for BERT when fine-tuning on various GLUE tasks: sequences corrupted at test-time by means of shuffling, sorting, duplicating, and dropping tokens still retain 70–90% performance of the non-perturbed input. Moreover, models appear to be as confident in assigning labels to perturbed inputs as they are to naturalistic ones. These results are corroborated by Pham et al. (2020), who show that models predominantly seek salient words in sequences, with numerous attention heads specializing themselves for this exact purpose. Sinha et al. (2020) report similar findings for various NLI datasets (in English and Chinese) across a variety of model architectures. They show that models are insensitive to word reorderings, some of which can actually result in improved task performance. Perhaps most strinkingly, Sinha et al. (2021) show that *pre-training* full-scale RoBERTa models on perturbed sentences (across n-grams of varying lengths) and fine-tuning them on unaltered GLUE tasks leads to negligible performance loss. They also report that a popular probe for dependency structure, that of Pimentel et al. (2020a), is able to decode trees from the perturbed representations—even a unigram baseline with resampled words–with considerable accuracy.

As a conceptual counterpoint to the permutation-based line of research, several studies have posed the opposite question: does explicitly injecting syntactic structure into models' representations or training objectives lead to better downstream performance? The observations in such studies are similar to the aforementioned work, albeit slightly more subtle: models that factor syntax into their decisions generally do not benefit in performance *via* its injection, which is taken to imply that such structure is redundant to the model, or not needed at all. Most notably, Glavaš and Vulić (2021) fine-tune BERT and RoBERTa (Liu Y. et al., 2019) as dependency parsers, before fine-tuning the same models again on NLI, paraphrase detection, and commonsense reasoning tasks. They show that, while intermediate parsing training (IPT) can produce near state-of-the-art parsers, repurposing these parameters for NLU tasks leads to negligible improvement. A similar trend is shown in Kuncoro et al. (2020), who train a BERT model distilled from an RNNG teacher (Dyer et al., 2016). They, too, find that, while their syntactically-aware model achieves top marks on a suite of parsing and otherwise syntactic tasks, the benefits for fine-tuning on GLUE are scant, if any. Swayamdipta et al. (2019) corroborate these findings for ELMo models conditioned on chunked input derived from phrase structure trees.

## 4. Discussion: A call for clarity and caution

After our general discussion of syntax, as well as our review of work exploring its role in contemporary language models, we are now in a position to make a few basic distinctions. In this section, we attempt to situate the findings of the aforementioned studies along several dimensions that we deem important toward the advancement of our research agenda.

### 4.1. Coding properties are not syntax

First, we would like to highlight the need to be clear about whether a study is concerned with abstract syntactic structure, overt coding properties, or with some relation between the two. A typical fallacy that may arise from not observing this distinction is to conflate a particular coding property with the abstract syntactic structure that it partially encodes. Naturally, if we fall victim to this fallacy when interpreting certain findings, we risk drawing conclusions based on insufficient or irrelevant evidence. This applies to situations where we may be tempted to employ coding properties as proxies of syntactic structure—either for attesting models' sensitivity to the latter or refuting it.

For example, it is important to acknowledge that studying agreement via TSE gives us a glimpse into how language models capture the syntactic relationship between selected words, such as verbs and their subjects. Per this view, high performance—even in the presence of various types of attractors—does not necessarily ential that a model has learned the grammar of a language. Rather, it has simply shown itself to be particularly sensitive to a single coding property, grammatical relation, or dependency type. Notably, English agreement is limited to expressing the number or person of the subject on the finite main verb (when in the present tense). This amounts to being, in the vast majority of cases, a binary distinction between correct and incorrect inflections, which bears a strong random choice baseline of 50% in the case of TSE. Thus, when one considers types of agreement manifested in other languages—such as number, gender, and case agreement between nouns and their modifying adjectives (e.g., German, Russian), or polypersonal agreement between a verb and multiple arguments (e.g., Basque, Georgian)—it becomes difficult to judge agreement as the primary mechanism by which syntax is encoded in English. Indeed, studies have shown that models tend to struggle with more expressive agreement mechanisms in morphologically rich languages (Ravfogel et al., 2018). Such insights call not only for a typologically driven research agenda, but also for nuance in interpreting positive findings for singular properties in selected languages.

We must also note that the above logic can apply in reverse: a model's lack of sensitivity to a single coding property, for example, word order (Dryer, 1992), does not imply that the model has failed to acquire syntax as a byproduct of its training objective. Even in a language like English, where word order is very salient, it is not the only coding property that signals syntactic structure. Consider *chases the cats the dog* as a permutation of *the dog chases the cats*: it is not unreasonable for an English speaker to decode the argument structure of this permutation using subject-verb agreement alone. Indeed, recent research in psycholinguistics has intimated that humans are relatively robust to permutations of linguistic form (Traxler, 2014). In the context of word order, Mollica et al. (2020) show that humans are able to process permuted sentences similarly to naturalistic ones, albeit when local structure (measured *via* pointwise mutual information) is preserved. Recently, this has been corroborated for models fine-tuned on GLUE as well, with performance therein strongly correlated with the extent of local structure corruption (Clouatre et al., 2022). With this in mind, one can see that order perturbation studies do not provide enough evidence to conclude that models (or humans) are insensitive to syntax. Instead, when conducting such studies, we must recall that word order (or agreement, for that matter) is simply a single coding property in a mosaic of such properties, all of which are privy to underlying processes that drive composition and comprehension.

### 4.2. Syntactic representations are not linguistic data

As a second point, if a study is concerned with syntactic structure, we need to clarify whether it assumes a specific type of syntactic representation, since the choice of representation may affect the results. Other things being equal, we may therefore prefer methods that do not presuppose specific syntactic representations, since conclusions will otherwise be valid only on the assumption that the chosen representation correctly captures syntactic structure. This consideration is even more important when we make use of automatically parsed data—as opposed to manually annotated sentences from treebanks—where otherwise sound syntactic representations may give misleading results due to parsing errors. At the same time, it is important to note that avoiding syntactic representations altogether may be limiting in another way, as it may restrict our methodological repertoire. Thus, as long as we maintain a critical attitude toward representation-dependent methods, they may still provide us with valuable results that cannot be obtained with other methods.

To illustrate the importance of representations in the context of probing, we can start by asking: does high UAS on a particular treebank imply that those trees are indeed *the* structures encoded by a given model? Or can alternative, linguistically plausible structures be decoded with comparable accuracy? Kulmizev et al. (2020) explore this question when probing various models for UD, a dependency formalism which prioritizes content-word heads (de Marneffe et al., 2021), and Surface-Syntactic UD, which assumes a traditional function-word head style analysis (Gerdes et al., 2018). They find that, while the difference in decoding UAS between the two formalisms is minimal for some treebanks, other treebanks exhibit strong preferences for either UD or SUD. They attribute such preferences to a complex interplay between the formalisms' inherent graph properties (e.g., average tree height), the probe employed for decoding (Hewitt and Manning, 2019's, in their case), annotation factors like tokenization, and morphology. Though preliminary, Kulmizev et al. (2020)'s study is a cautionary tale in tree-based probing, where choice of representation directly affects what conclusions one may draw about models.

We can ask similar questions when attempting to imbue models with syntactic structure. For example, is the injection of UD trees into a model's architecture enough to draw conclusions about the role of syntax in downstream performance? Or do alternative, linguistically plausible representations exist that models might yet benefit from? Beyond this, what privileges one particular injection method, say intermediate parsing training (Glavaš and Vulić, 2021), over another, such as knowledge distillation from an RNNG teacher (Kuncoro et al., 2020)? A template for exploring such considerations can be found in Wu et al. (2021), who report that infusing BERT with *semantic* dependencies can provide modest gains on GLUE. In that study, they compare the DM representation focused explicitly on predicate-argument structure (Ivanova et al., 2012) with the more syntactically oriented UD, finding that the former leads to slightly better performance^5^. Furthermore, they compare their chosen infusion method—semantic graph embeddings learned *via* a relational graph convolution encoder (Schlichtkrull et al., 2018)—with other means of injecting structure into representations, where their method performs best in most cases.

### 4.3. Data, model, and task

In any scientific pursuit, it is vital to acknowledge the (often vast) number of independent variables in play. For example, in studies concerning syntax in language models, we might acknowledge that our choice of model can be decomposed into various factors: architecture (Transformer, LSTM, etc.), pre-training task (auto-regressive or masked language modeling, infilling, etc.), pre-training data (size and domain thereof), model size, hyper-parameters, etc. Similarly, we might make considerations as how to source our experimental data (sampling corpora, grammar-constrained generation, crowd-sourcing, etc.) and how much of it to utilize. Indeed, it is not realistic to demand that future studies in this domain account for every aforementioned confound or enumerate all possible caveats. However, we nonetheless deem it vital for them to clearly articulate the interaction of data *D*, model *M*, and task *T* as it pertains to the particular aspect of syntax *A* that is in focus.

As noted earlier, this is the most easily done with TSE, where models are evaluated in their intended capacity (without an intermediary *T*), and *D* is employed as a representative sample of *A* (sourcing caveats notwithstanding). It is more complicated for probing studies for two reasons. First, although *T* can be a task related to syntax, *A* is typically not specified (outside a general notion of, e.g., tree structure). This becomes problematic when treating decoding accuracy as a measurement of the amount of syntactic knowledge in *M*'s representations, since the score is an aggregate dominated by easy, local constructions at the expense of more complex constructions that are more important from the point of view of hierarchical structure and compositionality. Second, the involvement of an intermediary probe *f* is a complication here, as it is not immediately clear whether syntax is actually encoded in *M*, or if it can be learned directly from *D*. Ravichander et al. (2021) demonstrate evidence of the latter, showing that *f* can “decode” *A* (verb tense, subject and object number, in their case) even if *M* was not exposed to any variation within *A* during pre-training (in other words, *M* had only seen, e.g., past-tense verbs). Although some proposals attempt to mitigate such confounds^6^, applying these methods requires researchers to conduct a survey of all such approaches and make a principled choice in employing one over another, which, we argue, centers *f* rather than the intended subject of study: *M*.

In downstream evaluation studies, the association between *D*, *M*, and *T* is yet trickier to disentangle. Similarly to probing, such studies entail fine-tuning the parameters of a pre-trained *M* on a separate task *T*, leading to an updated model *M*′. If *A* is considered, it is often with the goal of evaluating that particular aspect's importance in solving *T*, given a version of *D* that is corrupted accordingly (e.g., scrambled word order). Alternatively, syntax can be operationalized as a general notion (e.g., constituency structure) that is meant to inform *M* when performing across *T*. In either setup, *M*′'s performance on *T* (typically an NLU task like entailment) is typically attributed to *M*, where syntax is hypothesized as a necessity. Assembled this way, such experiments lead us to put our full trust in *T*, which we can employ as a prism through which to opine on *M*. This necessitates that *T* is indeed well-motivated and designed, and difficult to exploit *via* heuristics inherent to *D*—its attestation. Additionally, this presupposes that we possess a explanations of how humans employ syntax to solve *T* and that we can elicit comparative explanations from *M*.

If we believe the above to be true, we can hypothesize that, by performing well on such tasks, our models possess whatever latent ability humans do in solving them—see, e.g., Sinha et al. (2020): “models should have to know the syntax first, […] if performing any particular NLU task that genuinely requires a humanlike understanding of meaning.” Unfortunately, in the context of NLI (which Sinha et al., 2020 study) this is a highly dubious claim: the crowd-sourced nature of such datasets makes them prone to annotation artefacts (e.g., subsequence overlap between premise/hypothesis, lexical choice across inference classes, sentence length, etc.), which models often exploit as heuristics, thus leading to highly inflated performance metrics (Gururangan et al., 2018; Poliak et al., 2018; McCoy et al., 2019). Furthermore, though datasets for some tasks are supplemented with free-text rationales provided by annotators (Camburu et al., 2018; Rajani et al., 2019), self-rationalizing models introduce additional hurdles in terms of evaluation (what merits a model's rationale as being *acceptable*?) and interpretation (how *faithful* is a model's rationale to the label it generated?) (Wiegreffe et al., 2020; Jacovi and Goldberg, 2021).

Ultimately, the extent of trust we place in *M* (performing as hypothesized) over *T* (being correctly expressed) may influence not only our hypotheses, but also the conclusions we draw from our findings. For example, consider Pham et al. (2020) as a counterpoint to Sinha et al. (2020). Though the observations regarding BERT-based models' insensitivity to word order are largely similar, the former are more critical of the task (“GLUE does not necessarily require syntactic information or complex reasoning”), and the latter of the model (“current models do not yet ‘know syntax' in the fully systematic and human-like way we would like them to”). The interpretation of *M* is crucial here, as both studies are concerned with *M*′ (a textual entailment recognizer) rather than *M* (a language model). To this end, the accuracy-based performance of the former cannot, in principle, be used to interpret the syntactic knowledge of the latter, which is evaluated *via* different paradigms (perplexity or cross-entropy loss) and the weights of which have been overridden. By the same token, it is likewise important to avoid conflating a particular *M* (e.g., language models) with the architecture on which it is based (e.g., Transformers or LSTMs). This point is particularly salient if we consider work that targets models' inductive biases, which demonstrably shows that popular architectures often fail in making trivial, human-like generalizations (Lake and Baroni, 2018; Keysers et al., 2019; Lake et al., 2019; Gandhi and Lake, 2020; Kim and Linzen, 2020). It is therefore important to recognize that the success evinced, for example, in, TSE studies is a function of a neural network architecture applied specifically to a language modeling task, and that these results alone do not justify claims about the capacity of the architecture in general.

### 4.4. What are the research questions?

In addition to clarifying the role of data, model, and task in a given study, we also need to be clear about what our research questions are. For example, given a model *M*, a task *T*, a training dataset *D* and an aspect of syntax *A*, we may ask (at least) the following three questions:

1. To what degree *does M* learn *A* when trained on *D* to perform *T*?
2. To what degree *can M* learn *A* when trained on *D* to perform *T*?
3. To what degree does *M need* to learn *A* when trained on *D* to perform *T*?

Questions of type 1 are the most straightforward to investigate as long as we have a valid and reliable method for measuring the degree to which *M* learns *A* in the context of *D* and *T*. This is quite a big assumption in itself, and one that we will return to shortly, but we will focus first on the logic for answering different research questions. Questions of type 2 are modal in nature and therefore hard to investigate empirically, except indirectly by investigating questions of type 1. For example, in the pioneering study by Linzen et al. (2016), discussed in Section 3, the authors were primarily interested in whether an LSTM (*M*) can learn “syntax-sensitive dependencies” (*A*)—a question of type 2. To investigate this, they examined the actual learning behavior of the model in two specific settings (questions of type 1): (a) when trained on unlabeled text (*D*_*U*_) for the task of language modeling (*T*_*LM*_), and (b) when trained on labeled sentences (*D*_*L*_) for a specific agreement decision task (*T*_*A*_). The results were largely negative in the first case and positive in the second. From the positive result, they could conclude that the model *can* learn the relevant dependencies when trained on *D*_*L*_ for *T*_*A*_; from the negative results, they could however only conclude that there was no evidence that the model was capable of learning the relevant aspect of syntax when trained on *D*_*U*_ for *T*_*LM*_. This illustrates the fundamental asymmetry between positive and negative results when it comes to generalizations about possibility. A single positive result—if interpreted correctly—is sufficient to establish that something is possible, while any number of negative results are in principle inconclusive^7^. Indeed, as discussed in Section 3, the later study by Gulordava et al. (2018) managed to obtain positive results also in a setting similar to the first scenario of Linzen et al. (2016), from which they concluded that LSTMs are capable of learning at least one aspect of syntactic structure without explicit supervision. A similar conclusion was reached by Goldberg (2019) for the Transformer-based BERT model. The results are not directly comparable, because the latter study constructs the evaluation as a bidirectional masked language modeling task, but they are compatible in that none of the models have been trained with explicit syntactic supervision.

Questions of type 3 are more complex still, because they involve causality as well as modality. More precisely, they combine the question of whether learning *A* results in better performance of *M* on *T* (causality) with the question of whether learning *A* is necessary to achieve better performance (modality). A typical example is the study of Glavaš and Vulić (2021), discussed in Section 3, where the authors study the effect of intermediate parser training of a pre-trained language model later fine-tuned for various language understanding tasks. The underlying research question is whether knowledge of syntax is needed for language understanding—a question of type 3—and the lack of improvement may suggest a negative answer, but this conclusion is only warranted if it can also be shown (a) that the model has actually learned (some aspects of) syntax and (b) that this knowledge causally affects the model's behavior on the downstream task (and still fails to improve performance). Note, however, that a positive improvement would not be more conclusive in this case, because it would only show that improvement is *possible*, not that it is *necessary*. This illustrates the complexity involved when relating experimental results to research questions and points to the need for careful meta-analysis.

### 4.5. Aggregate metrics may be misleading—but are necessary

Let us finally turn to the question of how we can measure the degree to which a model *M* learns some aspect of syntax *A* when trained on data set *D* to perform task *T*—a question that is crucial to all studies in this area, regardless of what the more general research questions are. As we have seen in Section 3, the answer usually involves measuring performance on an appropriate task *T*′, although the exact solution depends on the type of study. In TSE studies, *T*′ is typically the task of discriminating positive from negative instances of some grammatical pattern, for example by assigning higher probability to the positive instance in a minimal pair. In probing, *T*′ is a supervised classification task assumed to reflect syntactic knowledge. And in NLU evaluation, *T*′ is simply the downstream language understanding task and thus normally coincides with the main task *T*. Each of these paradigms comes with its own methodological pitfalls, which have been extensively discussed in particular in the case of probing, but we will focus here on the complexities that are common to all of them.

First of all, we note that performance on *T*′ is almost always measured by averaging over individual test instances. In the simplest case, this may just be the arithmetic mean of a 0–1 loss metric, such as the accuracy reported for a probing classifier predicting part-of-speech tags. In other cases, it may be a more or less sophisticated macro-average, like an average over different grammatical patterns in a TSE study. In all cases, however, such aggregate measures need to be interpreted carefully. First of all, how do we know whether a given metric value indicates presence or absence of syntactic knowledge? Does a value of 0.5 mean that the glass is half full or half empty? This highlights the importance of relevant and informative baselines, a point that has been made in the literature before but that has perhaps not been fully appreciated. In addition, statistical significance tests should be used as appropriate.

Second, it is in the nature of aggregate metrics that they can easily be misleading by hiding important variation, especially if the distribution of different types of phenomena is heavily skewed. For example, in the related field of syntactic parser evaluation, Rimell et al. (2009) have shown that parsers with very respectable performance according to standard aggregate metrics like EVALB can have close to zero accuracy on certain types of unbounded dependency constructions. Moreover, aggregation may hide important variation in a number of different ways. If we use naturally occurring text in our test sets, certain words and constructions will inevitably be much more frequent than others and therefore dominate the aggregate scores in the same way as for syntactic parser evaluation. As a result of this, Newman et al. (2021) argue that standard metrics used in TSE overestimate the systematicity of language model behavior. If in addition we aggregate over different syntactic phenomena, we may hide the fact that different phenomena are captured to different degrees. And if we aggregate over multiple languages—or only report results for a single language—we may neglect important language-specific properties and risk over-generalization.

Lastly, we must consider the role aggregation plays in the interpretation of models' performance on benchmarks like BLiMP, SyntaxGym, or GLUE. At its core, such an enterprise entails that all aspects of syntax or language understanding—at least those of particular salience—have been successfully enumerated. Given the abstract nature of these notions, and the extent of debate regarding them, it is naturally doubtful that such an enumeration could ever be attained. Relaxing this somewhat, in assuming a salient set of aspects has indeed been collected, one must likewise assume—before aggregating—that a principled weighting of such aspects exists. This is especially relevant when dealing with a space of tasks or phenomena where fine-grained categorizations are likewise included—for instance, the six subject-verb agreement settings attested in BLiMP. In such cases, one must not only choose between micro and macro averaging across phenomena and their fine-grained attestations, but also articulate whether or not all phenomena lie on an equal playing field—in other words, that they are all equally (1) difficult to attest and (2) salient for evaluation. Certainly, in the vast majority of cases we assume a uniform weighting of classes when aggregating, since introducing hand-selected weights may introduce bias that we would otherwise prefer to avoid. However, we must not fail to acknowledge that benchmarks, in themselves, are influenced by designers' theories on what component parts adequately represent abstract notions like syntax or language understanding.

There is unfortunately no simple remedy to the complexities discussed in this section. In particular, giving up aggregate metrics is definitely not an option, since they are necessary for statistical significance testing and generalization. However, we believe that progress can be made by avoiding multiple aggregations and by making sure that we select our aggregate metrics to match our research questions and hypothesis. For example, if we want to know whether a model learns a general subject-verb relation, as opposed to memorizes agreement patterns for a small class of high-frequency verbs, then a macro-average over frequency classes will tell us more than a micro-average over all verb tokens.

## 5. Conclusion

The rapid progress in NLP thanks to deeper and larger neural network models trained on very large data sets with little or no linguistic supervision raises a number of questions concerning the status of traditional linguistic notions and theories in this landscape. Is there still a role for traditional techniques like supervised syntactic parsing? If not, is this because neural language models learn the relevant generalizations about linguistic structure without explicit supervision, or because language understanding does not really depend on such generalizations in the way traditionally assumed? If the latter, does this hold only for language understanding by machines, or does it also have implications for human language understanding?

These are exciting questions and it is therefore not surprising that we have seen a considerable body of research in this area recently. They are also difficult questions, and the methodology for tackling them is still under development, so it is also not surprising that results so far have been inconclusive and sometimes contradictory. As stated in the introduction, the goal of this article has not been to criticize previous efforts, but to contribute to our understanding of methods and results by articulating and discussing some of the inherent complexities in this research area. Without pretending to have any complete solutions, we want to conclude with some tentative conclusions and recommendations for future research, echoing the main points made throughout the paper.

Of the three approaches we have reviewed in Section 3, we are least optimistic about NLU evaluation, for several reasons. First, the research question that these studies address—whether syntactic knowledge is needed for a given task—is the hardest to tackle because it involves causality as well as modality. Second, it is often unclear what relation holds between the original pre-trained language model and its fine-tuned version. Last but not least, the whole endeavor is undermined by the uncertain status of current benchmark data sets when it comes to assessing language understanding. Taken together, these arguments appear to be fatal, and we think little can be salvaged from this approach.

When it comes to TSE and probing, we are slightly more optimistic, as long as a certain methodological rigor is maintained, as argued in Section 4. We need to be clear about what conception of syntax underlies our investigations, which aspects of syntax are being studied, and whether we make specific assumptions about syntactic representations. We need to explicitly discuss what research questions are being asked, and how they can be elucidated by the specific experiments we perform. We need to be careful when interpreting aggregate results, always looking for alternative metrics and additional analysis, and making sure to consider evidence from multiple languages if we want to draw conclusions about natural language in general. And we should in general resist the temptation to draw strong conclusions from any single study, which is usually impossible given the complex interplay of research questions, methodology, and data.

To make further progress, we also need to refine our methods of investigation. One way to do this is to combine different techniques in order to get a more complete picture of how a model processes a given type of phenomena. An obvious idea here is to combine probing and TSE, so that we can obtain systematic probing evidence related to specific phenomena, rather than aggregated over a heterogeneous test set, as is the typical practice today. A combination of techniques may also be used to bring downstream tasks back into the picture. In a recent study, Pérez-Mayos et al. (2021) uses structural probing, not to assess whether a single static model has learned syntax or not, but to track how syntactic capabilities evolve as a pre-trained model is fine-tuned for different tasks. One could imagine a similar experimental design using TSE instead of (or together with) probing. Another idea worth exploring is to increase the complexity of stimuli used for TSE or probing. The ability to produce and understand arbitrarily nested structures is a hallmark of compositionality and underexploited for analytical purposes.

Many researchers today seem to hold the view that, as language models get more and more powerful, their ability to learn syntax increases but the necessity to do so decreases for most tasks that we want them to handle. This may well be true, and maybe in this sense syntax *is* both dead and alive inside the black box. The evidence, however, is still far from conclusive, and we need more data as well as deeper analysis to make it so.

## Author contributions

All authors listed have made a substantial, direct, and intellectual contribution to the work and approved it for publication.

## Conflict of interest

The authors declare that the research was conducted in the absence of any commercial or financial relationships that could be construed as a potential conflict of interest.

## Publisher's note

All claims expressed in this article are solely those of the authors and do not necessarily represent those of their affiliated organizations, or those of the publisher, the editors and the reviewers. Any product that may be evaluated in this article, or claim that may be made by its manufacturer, is not guaranteed or endorsed by the publisher.

## Footnotes

1 In principle, the words of the Finnish sentence can be rearranged in any order without changing the syntactic roles, but some orders may be less natural and/or carry special pragmatic implications.

2 This term was coined, to the authors' best knowledge, by Marvin and Linzen (2018).

3 This suite was later named SyntaxGym in Gauthier et al. (2020).

4 Also known as diagnostic classification (see, e.g., Hupkes et al., 2018).

5 Interestingly, both syntactic and semantic models seem to outperform the fine-tuned RoBERTa baseline.

6 See, e.g., information-theoretic probing measures (Pimentel et al., 2020b; Voita and Titov, 2020; Hewitt et al., 2021), control tasks (Hewitt and Liang, 2019), or causal intervention (Elazar et al., 2021).

7 This is the mirror image of the case of necessity, underlying the famous quotation attributed (probably incorrectly) to Einstein: “No amount of experimentation can ever prove me right; a single experiment can prove me wrong”.
